# Present and future medical applications of microbial exopolysaccharides

**DOI:** 10.3389/fmicb.2015.01012

**Published:** 2015-09-29

**Authors:** Misu Moscovici

**Affiliations:** National Institute for Chemical Pharmaceutical Research and Development, BucharestRomania

**Keywords:** exopolysaccharides, medical applications, pharmaceuticals, perspectives

## Abstract

Microbial exopolysaccharides (EPS) have found outstanding medical applications since the mid-20th century, with the first clinical trials on dextran solutions as plasma expanders. Other EPS entered medicine firstly as conventional pharmaceutical excipients (e.g., xanthan – as suspension stabilizer, or pullulan – in capsules and oral care products). Polysaccharides, initially obtained from plant or animal sources, became easily available for a wide range of applications, especially when they were commercially produced by microbial fermentation. Alginates are used as anti-reflux, dental impressions, or as matrix for tablets. Hyaluronic acid and derivatives are used in surgery, arthritis treatment, or wound healing. Bacterial cellulose is applied in wound dressings or scaffolds for tissue engineering. The development of drug controlled-release systems and of micro- and nanoparticulated ones, has opened a new era of medical applications for biopolymers. EPS and their derivatives are well-suited potentially non-toxic, biodegradable drug carriers. Such systems concern rating and targeting of controlled release. Their large area of applications is explained by the available manifold series of derivatives, whose useful properties can be thereby controlled. From matrix inclusion to conjugates, different systems have been designed to solubilize, and to assure stable transport in the body, target accumulation and variable rate-release of a drug substance. From controlled drug delivery, EPS potential applications expanded to vaccine adjuvants and diagnostic imaging systems. Other potential applications are related to the bioactive (immunomodulator, antitumor, antiviral) characteristics of EPS. The numerous potential applications still wait to be developed into commercial pharmaceuticals and medical devices. Based on previous and recent results in important medical-pharmaceutical domains, one can undoubtedly state that EPS medical applications have a broad future ahead.

## Introduction

Exopolysaccharides (EPS) are extracellular carbohydrate polymers produced and secreted by microorganisms, which accumulate outside the cells. They are capable to be released into the surrounding environment. Despite their monomeric composition, similar to well-known plant or animal products, the EPS of different microbial origin (bacteria and fungi) display a large variety of structural combinations, which mediates them their unique properties. Microbial production shows several advantages over plant- or macro algae-derived products, such as defined and reproducible production parameters to circumvent environmental influences, and obtain a high quality of the final product. Additionally, much higher production titers can be obtained as compared to polysaccharides extracted from plants.

Obtained from easily available, renewable resources, biocompatible and apparently non-toxic, microbial EPS have found a very large field of applications, within which their medical uses play an important role.

This review study aims at achieving a useful insight in the domain, and at clearly distinguishing, in their historical development, the current commercial applications of EPS – officially acknowledged by worldwide accepted documents of medical authorities, from their promising potential applications – discussed in numerous publications. Such a valuable knowledge was updated and organized according to modern research directions in pharmaceutical science and therapy. The presented data highlight a real outlook and the necessary steps to enhance the efficiency and maximal exploitation of the scientific progress recorded in the EPS field up to date.

## Current Commercial Applications

Only a few microbial polysaccharides have found up to date commercial applications. Amongst them, **dextran**, a neutral polymer with α-(1→6) and α-(1→4) glucopyranosyl linkages was, discovered in wine in mid of the 19th century. Dextran could be considered the first remarkable example for a microbial EPS used in pharmaceutical applications (Nwodo et al., 2012). It was used as a plasma volume expander for controlling wounds shock since 1953 (Amspacher and Curreri, 1953; United States Pharmacopeia [USP] – National Formulary [NF], 2012; European Pharmacopeia [EP], 2014).

Further microbial EPS were employed in medical applications as pharmaceutical excipients, after they were approved as food additives. **Xanthan**, a bacterial branched anionic heteropolysaccharide composed of a five sugar repeating unit and different amounts of acetate and pyruvate, was discovered in 1950 (Born et al., 2002). Firstly it was used in large quantities for enhanced oil recovery, and later on was approved as a food additive in the USA (1969), by FAO/OMS (1974). In Europe, xanthan was approved as food additive as E415 (198 2), with subsequent inclusion in US and EU pharmacopeias (Born et al., 2002; United States Pharmacopeia [USP] – National Formulary [NF], 2012; European Pharmacopeia [EP], 2014). Similarly with food products, its properties as a thickener and suspension stabilizer are useful in pharmaceutical creams and suspensions and, recently, it has been used as a drug controlled release carrier (Morris and Harding, 2009).

**Alginate**, originally obtained by extraction from seaweeds, was discovered as a bacterial product in 1964, only differing from the seaweeds extraction product by the presence of acetyl groups in the linear structure of β-(1→4) mannuronic and α-(1→4) guluronic acid (Cyber Colloids Ltd.). This anionic polysaccharide is a good disintegrating agent in tablets (better than starch), a thickening and stabilizing agent in pharmaceutical suspensions and emulsions, as well as an antiacid stomach protector in capsules, as a sodium salt (Mc Hugh, 1987). Bacterial alginate has been also employed in cell microencapsulation, as microsphere vectors for drug delivery (Mukherjee and Atala, 2005; Nwodo et al., 2012). Five alginates were approved by FDA in 1973. Sodium alginate found its monographs in US and EU pharmacopeias (United States Pharmacopeia [USP] – National Formulary [NF], 2012; European Pharmacopeia [EP], 2014). Dental impression compounds are based on alginate cold-setting gels. Alginate fibers can be used as wound dressings and bandages with hemostatic properties, approved by FDA for human use (Mc Hugh, 1987; Mukherjee and Atala, 2005; Nwodo et al., 2012).

**Gellan** was discovered in 1978, as a bacterial anionic linear heteropolysaccharide, with a repeating unit of α-rhamnose, two residues of β-D-glucose and β-D-glucuronate. The native form contains acyl (acetyl and glyceryl) substituents. The acetyl groups can be easily removed by alkaline hydrolysis to lower the acyl content if necessary. Nineteen oral, 16 ophthalmic and six nasal drug formulations with a very large spectrum of therapeutic action are cited between 1993 and 2013 (Osmalek et al., 2014). Some of them became commercial medical products, determining the inclusion of low and high acyl forms of gellan in US pharmacopeia (United States Pharmacopeia [USP] – National Formulary [NF], 2012). Gellan has been FDA approved as a stabilizer and thickener in food since 1990. A low acyl form is used in solid dosage formulations, as a disintegration agent in immediate release tablets, or, in higher concentrations, as a matrix-forming excipient in sustained release, based on its swelling behavior (Osmalek et al., 2014; CP Kelco). In physiological ion concentrations, it forms *in situ* strong gels (Hagerstrom, 2003; Osmalek et al., 2014). In ophthalmic preparations, core gellan gum hydrogel showed a prolonged contact time (ocular residence) and enhanced bioavailability. Thus, ophthalmic controlled release anti-glaucoma preparations containing commercial low acetyl gellan are marketed under the trade name of Timoptic XE, or Blocadren depot (Merck, Co.; Felt et al., 2002; Hagerstrom, 2003).

**Pullulan** seems to be the single commercially produced EPS of fungal origin, with marketed pharmaceutical applications. It was discovered in 1938, but mostly studied after its description in 1959. This biopolymer is a neutral linear homopolysaccharide, consisting almost of regularly repeating α-(1→4) – maltotriosyl units (3-D-glucopyranosyl) joined through α-(1→6). Its present applications as a pharmaceutical ingredient are based on its distinct binding and film-forming properties, as well as on its strong oxygen impermeability. Such properties make it very suitable for granulation and coating tablets, non-animal capsules (Plantcaps-Capsugel, Inc.), oral and wound care products (e.g., Listerine – Pfizer; Mocanu et al., 2011b; Nagase Group; Tianjin SF–Bio). Approved firstly as a food ingredient in Japan (1976), USA-FDA (2002), Europe, as E1204, and China (2006), pullulan has been lately included in US, EU (United States Pharmacopeia [USP] – National Formulary [NF], 2012; European Pharmacopeia [EP], 2014) and Japan Pharmacopeias (Japan Pharmacopeia [JP], 2011).

**Hyaluronic acid** was discovered in 1934 and with its chemical structure established in the 1950s. Hyaluronic acid is an anionic linear polysaccharide belonging to the glycosaminoglycans, composed of disaccharide units consisting of (1→4) – β-linked-D-glucuronic acid and (1→3) – β-linked *N*-acetyl-D-glucosamine residues (Chong et al., 2005; Kogan et al., 2007; Necas et al., 2008). As sodium hyaluronate (hyaluronan), it plays important physiological roles in living organisms, including the human body. Its first medical application, maintained until now, was as a vitreous substitution/replacement during eye surgery in the late 1950s (Necas et al., 2008). Originally obtained by animal tissue extraction, especially from rooster coombs, it is now produced by recombinant bacteria. The bacterial product was firstly approved only for topical issues: chronic, difficult wound healing, e.g., Hyiodine, produced by Contipro (Contipro Pharma), and cosmetic applications. Lately, medical devices containing bacterial hyaluronan have been approved for use in eye surgery and intraarticular injections in osteoarthritis – e.g., Biolon – approved by Medical Device Certification-European Commission in 1995, FDA-USA in 1998, EUFLEXXA – approved by FDA-USA in 2011 (both produced by Ferring Pharmaceuticals – BTG), DUROLANE (EC-2004), produced by Bioventus (Bioventus). A sustained-release formulation of recombinant human growth hormone (SR-rhGH, DeclageTM, LG Life Sciences, Korea) using sodium hyaluronate microparticles was developed for administration on a weekly basis, being approved by Korean FDA in 2007. Recent clinical studies confirmed its efficacy and safety (Kim et al., 2014). Sodium hyaluronate has got a monograph in European Pharmacopeia (European Pharmacopeia [EP], 2014). A summary of the EPS mentioned above, including their medical applications is given in **Table 1**.

**Table 1:** Microbial exopolysaccharides with acknowledged commercial medical applications.

EPS	Monomer composition	Main producing microorganism	Applications	Reference
**Bacterial**				
Dextran	Glucose	*Leuconostoc mesenteroides*	Blood plasma volume expander (controlling wound shock)	Amspacher and Curreri, 1953; Nwodo et al., 2012; United States Pharmacopeia [USP] – National Formulary [NF], 2012; European Pharmacopeia [EP], 2014
Xanthan	Glucose (2), mannose (2), glucuronic acid, acetate, pyruvate	*Xanthomonas campestris*	Thickener, suspension stabilizer in pharmaceutical creams and suspensions Controlled release carrier	Born et al., 2002; Morris and Harding, 2009; United States Pharmacopeia [USP] – National Formulary [NF], 2012; European Pharmacopeia [EP], 2014
Alginate	Mannuronic acid, guluronic acid, acetate	*Azotobacter vinelandii, Pseudomonas aeruginosa*	Disintegrating agent in tablets; thickener, stabilizer in pharmaceutical suspensions and emulsions; dental impressions; antiacid (anti-reflux) stomach protector; microspheres for drug delivery; fibers in wound hemostatic dressing and bandages	Mc Hugh, 1987; Mukherjee and Atala, 2005; Nwodo et al., 2012; United States Pharmacopeia [USP] – National Formulary [NF], 2012; European Pharmacopeia [EP], 2014; Cyber Colloids Ltd.
Gellan	Glucose, rhamnose, glucuronic acid, glycerate, acetate	*Sphingomonas paucimobilis* (formerly *Pseudomonas elodea*)	Excipient in oral, ophthalmic and nasal drug formulations, for: tablet disintegration, sustained/controlled release	Felt et al., 2002; Hagerstrom, 2003; United States Pharmacopeia [USP] – National Formulary [NF], 2012; Osmalek et al., 2014; CP Kelco
Hyaluronic acid/hyaluronan	Glucuronic acid *N*-acetyl-glucosamine	*Streptococcus equisimilis*/*zooepidemicus; Bacillus subtilis* (recomb.*Str. equisimilis*)	Chronic, difficult wound healing; osteoarthritis treatment (intraarticular injection); eye surgery (vitreous substitution/replacement)	Kogan et al., 2007; Necas et al., 2008; Food and Drug Administration-Premarket Approval Application [FDA-PMA], 2011; European Pharmacopeia [EP], 2014; Bioventus; Contipro Pharma
**Fungal**				
Pullulan	Maltotriose	*Aureobasidium pullulans*	Tablet granulation and coating, binder, and oxygen impermeable film forming, non-animal capsules, oral, and wound care products	Japan Pharmacopeia [JP], 2011; Mocanu et al., 2011b; United States Pharmacopeia [USP] – National Formulary [NF], 2012; European Pharmacopeia [EP], 2014; Nagase Group; Tianjin SF–Bio

## Potential Applications

An impressively large volume of publications have been dedicated to the potential medical applications of EPS, considering not only their number, but also their diversity. The present paper aims at grouping such works in an accessible manner, to obtain a relevant and suggestive image of the field.

### Drug/Hydrogel Controlled Release Systems

#### Micro- and Nanosystems for Sustained Delivery

Polysaccharides and, among them, EPS, have become highly promising materials in the field of “intelligent drug delivery systems” or “intelligent therapeutics” (Caldorrera-Moore and Peppas, 2009; Patel et al., 2011). The ability of such hydrophilic polymers to form hydrogels, cross-linked 3D network structures retaining a large amount of water while remaining insoluble, makes them very useful as drug carriers. Thus, as macro- and especially nanoparticles, such gels can entrap drugs or biomolecules into their inner structures and/or adsorb the therapeutic molecules onto their external surfaces and penetrate cells and tissue gaps to arrive at target organs. Having reached them, the drug delivery systems can show different drug rate-release properties due to their various characteristics, including bioadhesion, biodegradability, pH, ion and/or temperature sensitivity. In this way, they can prolong drug residence and, therefore, its therapeutically usable fraction, thus increasing its bioavailability and permitting lower doses with consequently reduced toxic effects (Liu et al., 2008). Natural polysaccharides have a large number of reactive groups, a wide range of molecular weights and different chemical composition, leading to highly diverse structures and properties; additionally, they are biodegradable, non-toxic and safe.

##### Hydrogels with thermo-, pH-, and cation-sensitive drug-delivery properties

Pullulan has been intensively studied as a drug carrier in pharmaceutics, particularly because of its neutral nature with nine hydroxyl groups on the repeating unit conferring to it a special availability to chemical derivatization. By cross-linking and grafting on the backbone, poly-(*N*-isopropyl-acrylamide)-co-acrylamide and ether succinic carboxyl groups, thermo- and pH-sensitive microspheres were obtained. With lysozyme as the protein drug model, both simple and electrostatic (ionic) entrapment and pH/thermo sensitive release were achieved (Fundueanu et al., 2008). Pullulan/silver nanoparticles composite nanospheres with controlled spherical structure showed to enhance antibacterial activity (Kumar et al., 2012). Silver-containing nanoparticles have been also obtained by partial oxidation of pullulan (carboxylation; Coseri et al., 2015). Carboxymethyl pullulan, grafted with polyether-amine, as the thermo sensitive part of the copolymer, exhibited a sol-gel transition at body temperature, assuring sustained drug-delivery (Dulong et al., 2012). Such polymers, containing carboxymethyl pullulan, cross-linked to thermo- and pH-sensitive hydrogels, gradually retained the antioxidant biomolecules – lutein and α-tocopherol – and showed scavenging activity (Mocanu et al., 2012). *In situ* rapid cation-induced gelation of gellan favored, the epithelial uptake (at a residence time shown *in vivo* as long as 4 h), and transfer of a model substance demonstrated the use of gellan as a promising strategy for nasal drug delivery, thus avoiding a slow and reduced absorption of some drugs by oral administration (Hagerstrom, 2003).

##### Amphiphilic EPS, as controlled delivery systems

Grafting of hydrophobic segments onto the hydrophilic polymeric backbone leads to amphiphilic polymers which form self-associate thermodynamically stable nanogel structures, with an inner hydrophobic core. Stable in size over time, such polymeric micelles have been recognized as promising drug carriers, the hydrophobic core-shell structure being able to trap hydrophobic drugs (Liu et al., 2008; Bataille et al., 2011; Jiang et al., 2013). In this respect, the most cited paper was an early work from 1993 on the self-assembly of cholesteryl-bearing pullulan (CHP), forming stable hydrogel nanoparticles, which represented the starting point for future perspective drug-release complexes (Bataille et al., 2011). Thus, self-aggregated nanoparticles of cholesterol-modified pullulan with succinyl linkages were loaded with mitoxantrone (as model anticancer) and exhibited sustained release (Yang et al., 2010).

Other cross-linking microspheres obtained from carboxymethyl pullulan became more adsorptive by their ionic and hydrophobic affinity for lysozyme. After hydrophobization with palmitoyl the release rate was also controlled (Mocanu et al., 2004).

##### Interpenetrating polymer networks (IPNs)

Interesting potential medical applications may be realized by the hydrogels of polysaccharides. Such “smart” systems, consisting of penetrating polymers at molecular scale, promise to be perspective drug-carriers. Thus, interpenetrating polymer network (IPN) microspheres of alginate and synthetic or natural polymers showed entrapping and sustained release properties of different drug substances: anticancer 5-fluorouracyl, anti-inflammatory indomethacin, antibiotic gatifloxacin, anticoagulant heparin, NSAID sulidac, as well as intestinal release of the poorly soluble anti-hypertensive pindolol (Matricardi et al., 2013).

Beads of gellan gum/pectin mixture, obtained by ionotropic gelation, using Al^3+^ as crosslinker, showed suitable entrapment and mucoadhesive properties for enteric *in vitro* controlled release of anti-inflammatory ketoprofen (Prezotti et al., 2014).

Poly (ε-caprolactone)-grafted dextran nanoparticles entrapped and exhibited *in vitro* sustained release of amoxicillin antibiotic (Saldias et al., 2015).

Steroid hormones, as important drug-substances, whose administration may be improved by sustained delivery, are almost absent in latest EPS research. Micro- and nanoparticles containing such compounds in transdermal drug delivery systems can avoid many of the side effects associated with first-pass metabolism, gastrointestinal absorption and high plasma levels after oral administration, reaching clinically significant concentrations at lower doses. Studies on their pharmacokinetics, bioequivalence, local tolerance, and adhesion performance are necessary.

Another suggestion involves grafted block copolymers of only EPS, not considered in recently published research results.

#### Drug-Targeting, Macromolecule, and Cell Carriers

##### Drug-targeting

Polysaccharide conjugates, especially the amphiphilic ones, with different hydrophobization degrees, showed particular binding and penetrating features to cellular receptors, appearing as promising drug-targeting carriers.

Thus, cholesteryl-pullulan nanoparticles can hydrophobically interact with the beta oligomer forms of beta-amyloid, significantly reducing its toxicity, which appears as a possible complementary approach in neurologic disorders with formation of soluble toxic aggregates, e.g., in Alzheimer disease (Boridy et al., 2009).

##### Anticancer drug-targeting

Self-assembled nanoparticles of carboxymethyl curdlan, a known (1→3)-β-glucan, hydrophobized by deoxycholic residues, physically loaded with anticancer epirubicin, increased the drug uptake in tumors and decreased it in kidney and heart on tumor-bearing mice, showing a sustained release pattern and a tumor volume reduced by 70% (Gao et al., 2010). Other hydrophobized polysaccharide – bile acid conjugate (hyaluronic-cholanic acid) nanoparticles showed a receptor-mediated *in vitro* and *in vivo* preferential accumulation, in murine carcinoma SCC7 cells (Choi et al., 2009). Hyaluronic acid-coated solid lipid (freeze-dried) nanoparticles showed *in vitro* and *in vivo* a receptor-mediated, sustained and targeted delivery of anticancer vorinostat to SCC7 carcinoma, human breast adenocarcinoma (MCF-7) and human lung epithelial adenocarcinoma (A549; Tran et al., 2014).

Recently, a novel gellan nanohydrogel system was developed, for simultaneously carrying two poorly water soluble drugs (physically entrapped paclitaxel and chemically linked prednisolone) to achieve target delivery of such anticancer and anti-inflammatory drugs. Promising *in vitro* results – induced by their synergistic effect – on three types of cancer cells, were noticed (D’Arrigo et al., 2014).

Transdermal drug delivery is an important pharmaceutical alternative for drug-targeting in skin diseases. Hyaluronic acid nanoemulsions showed an efficient selective *in vitro* and *in vivo* controlled-release of methylene-dioxycamptothecin, an inhibitor of keloid dermal tumors (Gao et al., 2014).

Curcumin-loaded hydrogels of xanthan-plant galactomannan mixture were effective in skin-diseases, as anti-inflammatory and antioxidant drugs, which may recommend them as a promising alternative in the treatment of skin-cancer and psoriasis (Koop et al., 2015).

Biolabile prodrug compounds (releasing the drug by micro flora enzymatic glycosidase hydrolysis of the gels selected from dextran, pullulan, and alginates), or cross-linked dextran hydrogels showed colon-specific drug delivery (Vandamme et al., 2002). Another prodrug conjugate, the hyaluronic acid-methotrexate, an anti-inflammatory substance, optimized the treatment of osteoarthritis, showing *in vivo* effect on rats (Homma et al., 2010).

A very promising, still non-explored field is the use of properly functionalized EPS as drug-carriers, to cross the blood-brain barrier for treating tumors or other neurological diseases.

##### Recombinant macromolecular biopharmaceuticals

Proteins, peptides, small-interfering RNA (siRNA), vaccines and hormones – represent a rapidly growing class of modern therapeutics, superior to small drugs for serious and deadly diseases, as well as for diagnostics. However, they have difficulties in crossing mucosal surfaces and biological membranes, due to their susceptibility to lose the native structure and to be rapidly cleared in the liver or other body tissues. Polymeric hydrogels used as carriers could diminish their instability and improve bioavailability, permitting other routes of administration, apart from the frequently injectable: pulmonary, oral, nasal ones (Ganguly et al., 2014).

In this respect, special importance is paid to insulin, a long-term treatment drug of *diabetes mellitus*. Nanoparticles of cholesterol-bearing pullulan preserved insulin from enzymatic degradation, its activity being unchanged after i.v. injection (Akiyoshi et al., 1998). Insulin was 85% electrostatically associated to nanoparticles of dextran sulfate/chitosan, with a 24 h sustained release in a simulated intestinal medium, suggesting a possible oral delivery (Sarmento et al., 2006). Vitamin B12-derivatives-coated dextran nanoparticles encapsulated more than 65% insulin and demonstrated a prolonged hypoglycemic action on diabetic mice and rats, after oral administration (Chalasani et al., 2007).

Recombinant human growth hormone-Zn^2+^/dextran nanoparticles preserved 99% of hormone bioactivity (Yuan et al., 2012). Lysozyme, as a model protein, was highly retained and well-released on pH and thermo sensitive (Poloxamer grafted) carboxymethyl pullulan micro particles (Mocanu et al., 2011a).

The hyaluronic acid-gold nanoparticle/interferon α complex showed target specificity and prolonged delivery in the murine liver tissue, leading to superior immune responses than the known interferon α preparation for the treatment of hepatitis C virus infection (Lee et al., 2012). Nowadays, successful non-interferon therapeutics diminishes the importance of such systems.

##### Vaccines

Exopolysaccharide could be useful as antigen-carriers or as antigens themselves in vaccine preparations. Tetanus toxoid (anatoxin) was entrapped and efficiently released on Poloxamer hydrophobized carboxymethyl pullulan micro particles (Mocanu et al., 2011a). Intranasal immunization with alginate-tetanus toxoid microparticles resulted in a strong immunoresponse in rabbits. Esters of the hyaluronic acid loaded with hemagglutinin influenza H1N1 were also effective after intranasal administration in mice, rabbits, micro-pigs (Sharma et al., 2009). Curdlan sulfate enhanced antigen-specific immunity in mice immunized with human recombinant hepatitis B protein, appearing as a promising vaccine adjuvant (Li et al., 2014). Purified by ultrafiltration, type B capsular polysaccharide produced by *Haemophilus influenzae*, which was linked to a protein, became a component of polyvalent vaccines against severe infections in children (e.g., meningitis; Albani et al., 2015; De Oliveira Cintra and Takagi, 2015).

As novel adjuvant systems, EPS could enhance vaccine-induced protection, providing a strong tailored and immune response, especially targeting challenging pathogens, such as new influenza pandemic strains (e.g., H1N1, parasites (malaria), highly variable viruses (hepatitis C, AIDS), or resistant mycobacteria (tuberculosis). Another direction is represented by antigen-specific cancer immunotherapy, stimulating an immune response to reject the tumor.

##### Gene delivery

Non-viral vectors, e.g., polymers, are preferred to deliver nucleic acid materials, to improve the transport and avoid degradation by lysosomal enzymes. Plasmid encapsulation in pullulan nanoparticles demonstrated a successful internalization (Gupta and Gupta, 2004). Cationic dextran derivative nanoparticles, loaded with gene silencing siRNA, achieved an effective transfection in hepatoma Huh-7 cells, by association with a photosensitizer (Raemdonck et al., 2010). The same genetic material was also transferred by PEG-ylated dextran to Huh-7 and A 431 human epithelial carcinoma cells (Naeye et al., 2010). Cationic siRNA loaded 6-amino-deoxicurdlan efficiently transfected human lung H727 and human colon HCT116 cancer cell lines, human leukemia monocyte THP-1 cell-derived macrophages, as well as mouse primary and stem cells (Han et al., 2015). Other gene-delivery studies, including pDNA with EPS derivatives, mostly amino-modified, e.g., gellan, alginate, schizophyllan and scleroglucan, fungal β-(1→3)-glucans with β-(1→6)-glucose side chains, were also cited (Khan et al., 2012; Zhang et al., 2013).

Using EPS as gene delivery vectors is a recent potential application which should be confirmed by *in vivo* studies, as future research depends on gene therapy development in cancer.

##### Cell encapsulation

This technology is considered protecting transplanted cells from hostile immune reactions of the body, assuring at the same time the permeation of nutrients and secreted proteins. The transplantation of encapsulated cells has been considered a promising treatment for a variety of diseases (e.g., diabetes, liver failure; Rokstad et al., 2014). To make able the 3D structure of bacterial cellulose (BC) nanofibers to form a cross-linked alginate-based composite with improved mechanical and chemical stability, BC, produced by *Gluconacetobacter xylinus* fermentation, was oxidized at C-6 in the NaBr/NaClO/TEMPO (tetramethyl-piperidine-1-oxyl) system as a catalyst. The C-6-carboxyl-cellulose-sodium alginate beads successfully encapsulated fibroblast cells. Such composite is considered a candidate to encapsulate cells forming islets to activate insulin secretion (Park et al., 2015).

Obtaining encapsulated cells within a three-dimensional hydrogel layer is considered as a new direction in microscale tissue engineering (Evans et al., 2006).

#### Wound Healing and Tissue Engineering

##### Wound healing-skin repair

###### Bacterial cellulose

Bacterial cellulose is produced by bacterial fermentation with controllable 3D structure, based on strains selection or cultivation parameters (Lin et al., 2013). The resulting non-water soluble nanofibril network shows high similarity to that of collagen and has an outstanding biocompatibility (Torres et al., 2012). The applications of BC are in the field of wound healing-skin repair, as absorbent of exudates as well as permeable material. Compared to plant cellulose, BC possesses a high crystallinity, a high water absorption capacity, as well as high resistance to microbial or enzymatic degradation. Its properties as a wound healing and tissue engineering scaffold could be diversified and improved according to specific aims, by chemical derivatization or association of other synthetic, mineral (e.g., hydroxyapatite) substances, biopolymers (e.g., hyaluronan, alginate, gellan, carrageenan, chitosan, collagen, gelatin, elastin), or cell-growth factors (Torres et al., 2012; Fu et al., 2013). Clinical trials with BC-dressings on acute and chronic wounds (e.g., diabetic foot ulcers) showed superior results than similar dressings from plant-cellulose (Fu et al., 2013). BC-glycerin membranes with a clinically proved moisturing effect could be relevant for dryness induced by skin diseases, such as psoriasis and atopic dermatitis (Almeida et al., 2014). Electrospun acetylated BC presented a more symmetric nanopore structure than that of the casting films, suitable for cell integration and adhesion (Costa et al., 2012). Dressings with hydrogels of BC/acrylic acid synthesized by electron beam-irradiation accelerated burn wound healing in rats (Najwa et al., 2014).

##### Tissue engineering

Heart tissue engineering aims at designing structures to support, repair, replace, or enhance the function of injured or diseased myocardial tissues, especially as caused by infarction. In this respect, alginate, pullulan, dextran, hyaluronan have been intensively studied (Silva et al., 2015). Photopolymerizable hyaluronic acid-methacrylic anhydride (HA-MA) hydrogels mimicked the extracellular matrix in heart valve applications (Aravamudhan et al., 2014). Alginate-based hybrid copolymers with poly(propylene) fumarate, morphologically modified by covalent linking with acrylates, acquired improved mechanical properties. One of them was described to promote cardiomyoblast growth, recommending it for potential applications in cardiac tissue engineering (Thankam and Muthu, 2014).

Schiff-base formation between oxidized dextran (aldehyde) and poly-L-lysine led to a potential bioadhesive in surgery (Matsumura et al., 2014). Carboxymethyl pullulan-heparin conjugates were developed and studied for tissue engineering applications (Mishra et al., 2011). Electrospun gelatin nanofibers cross-linked with oxidized dextran (aldehyde) demonstrated good scaffold properties for L-926 fibroblasts (Jalaja et al., 2014). Alginate scaffolds were effective for chondrocyte culture stem cells, as a promising solution for cartilage regeneration, combating osteoarthritis and arthroplasty. If they contain chitosan, calcium, or are impregnated with antibiotics, they could enhance the antibacterial and wound healing effect of bandages (Ivanova et al., 2014). Alginate materials have been successfully used in tissue-engineering of bioartificial pancreas, bone, vasculature, and liver cell cultures (Aravamudhan et al., 2014). Some new EPS produced by bacteria isolated from extreme marine environments showed promising properties for tissue engineering in bone healing (Mancuso Nichols et al., 2005).

In tissue engineering, special interest is paid to stem cells and the design of bioactive nanopatterned scaffolds of different polymeric materials, including EPS, with specific ligands that direct and enhance cell function and differentiation of embryonic stem cells (Evans et al., 2006).

### Diagnostics

Polysaccharide-coated nanoparticles used in diagnostics (e.g., quantum dots, magnetic materials, such as iron oxide) could play a key role in medical imaging and also in theranostics (diagnosis and therapy). Specific ligand groups could be attached to the biopolymer, such as amino. Cholesterol pullulan modified by amino groups showed a higher intensity of fluorescence in tumor cells, comparatively with conventional quantum-dots-liposomes (Bataille et al., 2011; Mishra et al., 2011; Prajapati et al., 2013). A complete platform of super paramagnetic iron oxide nanoparticles with cross-linked dextran coating (CLIO) – containing large series of multifunctional imaging agents for diagnostic magnetic resonance (DMR), magnetic resonance imaging (MRI), positron emission tomography (PET) imaging, fluorescence molecular tomography (FMT) – has been developed. As a theranostic agent, CLIO could be used with a photosensitizer in photodynamic therapy, killing atheroma cells in carotid arteries through irradiation (Tassa et al., 2011). A theranostic effect was obtained with acetylated pullulan-coated magnetic nanoparticles, killing 80% of the KB tumor cells by magnetic field-induced hyperthermia (Bataille et al., 2011; Mishra et al., 2011).

Theranostic application of such diagnostics implies repeated dose administration, depending essentially on the metabolism and elimination of iron particles.

### Bioactive EPS as Potential Therapeutics

#### Fungal β-Glucans

Some EPS showed biological activities which promote them as potential therapeutics. A special interest in this view has attracted β-glucans. Generally, the immunomodulation effect of β-glucans is due to their interactions with macrophage receptors, activating these cells as basic effectors in host defense against bacteria, viruses, parasites, and tumor cells (Novak and Vetvicka, 2009). Therefore, from the early 90s on, the backbone of β-(1→3) glucans has been considered as essential for their antitumor activity, based on the Sarcoma tumor inhibition by sulfoalkyl-curdlan (Demleitner et al., 1992), as well as for their immunopotentiating, antibacterial, or antiviral activity (Kulicke et al., 1997). β-glucans have shown varying activity against sarcomas, mammary cancer, some chemically induced cancers, colon cancer, and some leukemia (Laroche and Michaud, 2007).

##### Schizo- and scleroglucans

The most important representatives of fungal beta-glucans are schizophyllan (SPG) and scleroglucan (SG), neutral EPS produced by *Schizophyllum commune* (Zhang et al., 2013) and *Sclerotium rolfsii*, respectively (Survase et al., 2007). They are composed by a β-(1→3)-D-glucopyranose backbone which is branched with a single β-(1→6)-D-glucopyranose residue at every third glucose unit. Single or associated with chemotherapeutics, these EPS and their derivatives showed promising antitumor (in sarcoma, carcinomas, bladder tumor, fibro sarcoma, mammary carcinoma, leukemia) and immunopotentiator activities. SPG has been approved for clinical studies in Japan (Daba and Ezeronye, 2003; Zhang et al., 2013). SSG, produced by the *Sclerotinia sclerotiorum* fungus, whose structure is similar to that of schizophyllan, yet more branched (a glucose residue occurs at every two β-1, 3-glucosyl units) showed antitumor activity on Sarcoma 180 in mice (Ohno et al., 1986).

The β-(1→3) backbone, as mentioned above, but the β-(1→6)-glycosidic linked branches structures, too, appear as important for antitumor activity.

##### Botryosphaeran

Represents another β-(1→3, 1→6)-glucan (1→3 backbone, 1→6 branched glucose and gentiobiose) and is produced by *Botryosphaeria rhodina*. Botryosphaeran showed anticlastogenic activity *in vivo* (mice) after cyclophosphamide (Miranda et al., 2008). It also exhibited antidiabetic (reducing plasma glucose level in streptozocin-induced diabetic rats by 52%) and hypocholesterolemic activities (total and LDL cholesterol reduced until 27% in hyperlipidemic rats; Miranda-Nantes et al., 2011). Chemical modifications increased biological activity (Kagimura et al., 2015). The sulfonated derivative induced *in vitro* a dose-dependent anticoagulant and antithrombotic activity (Mendes et al., 2009).

##### Lasiodiplodan

Exopolysaccharide, a β-(1→6)-D-glucan produced by *Lasiodiplodia theobromae*, showed anti-proliferative activity in breast cancer MCF-7 cells (Alves da Cunha et al., 2012), whereas its sulfonated derivative exhibited anticoagulant and antithrombotic activity similar to that of heparin (Vasconcelos et al., 2013).

A hetero-EPS (mainly mannose, glucose, galactose, xylose) produced in submerged cultivation of the *Pycnoporus sanguineus* fungus exhibited *in vitro* antioxidant activity (Cao et al., 2014). Similar results were obtained with a *Hirsutella* sp. fungus (neutral EPS containing mannose, glucose, galactose), proving the importance of the mannose content (Meng et al., 2015). A fraction of α- and β-EPS (mainly composed of mannose, glucose, and talose), produced in a submerged culture by the Chinese medical fungus *Inocutis tamaricis*, exhibited *in vitro* antioxidant and antitumor (Hep G2 cells) activities (Zheng et al., 2014).

Antitumor activity of EPS is immune-mediated (immunotherapy) by the activation of the T and B defender cells against cancer cells. Although their action is slower than that of traditional therapies (chemotherapy, radiation), it is nevertheless specific, adaptable and durable. Thus, immunotherapeutic EPS could be valuable prophylactic and synergic anticancer agents.

#### Bacterial EPS

##### Lactic bacterial EPS

One group of bioactive EPS is represented by those produced by lactic bacteria (LAB). A 2-substituted β-(1→3)-D-glucan produced by *Pediococcus parvulus* 2,6 and a recombinant *Lactococcus lactis* showed immunomodulation by human macrophage activation *in vitro*, promoting the production of anti-inflammatory cytokines (Notararigo et al., 2014). A similar effect was noticed with a neutral hetero-EPS (glucose, rhamnose, galactose) produced by a *Lactobacillus paraplantarum* strain in an *in vitro* study as a probiotic agent (good adherence to intestinal mucosa; Nikolic et al., 2012). Other EPS of LAB species (e.g., *Weissella cibaria*), isolated from the gastrointestinal tract of marine fish, showed a prebiotic *in vitro* activity, stimulating probiotics (e.g., bifidobacteria; Hongpattarakere et al., 2012).

A β-D-(1→4) with 1:2 β-D-(1→6) branched glucan produced by an isolated Chinese *Rhizobium* showed immunopotentiating and antitumor activities in mice with sarcoma 180 (S180), hepatoma 22 (H22) and Ehrlich ascites carcinoma (EAC; Zhao et al., 2010).

##### Bioactive xanthan derivative

Xanthouronan, obtained by C-6 oxidation of xanthan with a NaOCl/NaBr/TEMPO catalyst, exhibited *in vitro* antioxidant activity (Delattre et al., 2015).

Hetero-EPS produced by nature isolated *Enterobacter* bacteria strains, composed of fucose, glucose, galactose, glucuronic acid, pyruvate, succinate, acetate (Freitas et al., 2011b; Huang et al., 2015) showed hypoglycemic and hypolipidemic activities in type 2 diabetic mice (Huang et al., 2015). The signaling pathways of action and the corresponding biomarkers – still under study – are probably similar to plant extraction active polysaccharides.

**Levans** represent an EPS group of β-(2→6)-D-fructans with some β-(2→1)-branches synthetized from sucrose by levansucrase, produced by several bacteria, including species of *Bacillus*, *Zymomonas*, *Halomonas*, *Pseudomonas*, *Rahnella*, *Aerobacter*, *Erwinia*, *Streptococcus*, *Microbacterium* (Freitas et al., 2011a; Esawy et al., 2012; Öner, 2013; Srikanth et al., 2015). The levan produced by a *Halomonas smyrnensis* strain, especially its oxidized (aldehyde) derivative, showed *in vitro* anti-cancer activity against human lung (A549), liver (Hep G2/C3A), gastric (AGS), breast (MCF-7) cancer cell lines (Sarilmiser and Öner, 2014). Acetyl, phosphoryl, and benzyl esters of levan EPS, produced by a strain of *Paenibacillus polymyxa*, exhibited antioxidant and anti-proliferative activities against human gastric BGC-823 cancer cells *in vitro* (Liu et al., 2012; Zong et al., 2012). The levan produced by *Bacillus licheniformis* exhibited hypoglycemic and antioxidant activities, enhanced enzymatic defense, protecting the main organs in alloxan-induced diabetic rats (Dahech et al., 2011). The levans produced by honey isolated *Bacillus subtilis* strains showed antiviral activity on avian influenza and type 40 adenovirus (Esawy et al., 2012). The anti-AIDS activity of levan was also noticed (Srikanth et al., 2015).

Antiviral activity, enhanced by sulfation, of other bacterial EPS, was also noticed earlier (Matsuda et al., 1999; Laroche and Michaud, 2007), as due to the interference with viral attachment and penetration/infection of cells, probably by interacting with viral envelope glycoproteins. Recent results, obtained by surface plasmon response technology, confirmed the interaction, curdlan sulfate binding the recombinant human hepatitis B viral protein, with increased affinity toward the sulfation degree (Li et al., 2014).

## Perspectives/Conclusion

Microbial EPS offer a very large field of medical applications, increasingly exploited, yet at a too slow rate, if considering their obvious advantages, some of them induced by their unique properties.

As to the solid dosage forms and rate-controlled release systems, they compete with cheaper natural polymers, as plant cellulose and starch derivatives, specific niches (intelligent therapeutics) being still to be established, along with their cost-effectiveness, by means of new, performant technologies.

As drug-targeting and carriers, EPS nanoparticulate systems present certain advantages over those of chemical synthetic origin, such as biocompatibility and apparent lack of toxicity. However, they are not excluded from the mandatory non-clinical pharmacotoxicology studies, as the chemical modifications undergone by natural EPS still have to be carefully analyzed. In this stage of research, the most important challenge, common to all nanopharmaceuticals, is to demonstrate their *in vivo* bioavailability for assuring a suitable drug exposure, by passing from the discovered particular effects to well-established research protocols of absorption, distribution, metabolism and elimination (ADME), drug metabolism and pharmacokinetics (DMPKs). In this respect, recent developments in EPS nanoparticle medical imaging systems could be very useful, having also higher chances to be approved as diagnostics. Obviously, based on all the above-mentioned considerations, the external administration forms with topic action (e.g., wound healing, skin-repair) are likely to be more rapidly approved for and to pass clinical trials, as well as to enter the market, followed by EPS-nanopharmaceuticals which have already shown *in vivo* promising results, as well as by those which avoid the digestive way (e.g., transdermal, nasal).

The EPS nanoparticulate systems should exploit some of their advantages over liposomes (e.g., higher stability and versatile functionalization), which have already advanced in approval of clinical trials. More research effort in specific challenging areas of high interest, e.g., brain-targeting in neurological disorders (stroke, tumors, Alzheimer’s), is expected.

Regarding bioactive EPS as potential therapeutics, their challenge is to prove, necessarily *in vivo*, therapeutic advantages over the drugs available on the market, and to observe the above-mentioned well-established protocols for new drugs. Research on associative formulations with already known therapeutics for synergetic effects should be also considered. New studies on the molecular biology mechanisms of action will highlight structure-activity relationships.

Nevertheless, the important therapeutic domains having recorded promising results (e.g., cancer, diabetes, vaccines) still expect further intense world-wide research work and a higher involvement of microbial EPS in health improving resources.

## Conflict of Interest Statement

The author declares that the research was conducted in the absence of any commercial or financial relationships that could be construed as a potential conflict of interest.
